# Mental Disease and Mental Death

**Published:** 1907-04-13

**Authors:** 


### MENTAL DISEASE AND MENTAL DEATH.

### IS THE PRESENT ASYLUM SYSTEM A FAILURE?

### I. The Existing Position.

Insanity occupies a very special and peculiar position in medical practice. It is only during the last few years that it has had a position in the medical curriculum, so that the vast majority of the older practitioners have had no opportunity whatever of acquiring a systematic knowledge of it. Since the study of insanity has been compulsory on medical students, the number and urgency of other claims have been so great that the time given to the study of insanity has been so short, and the opportunities of seeing cases so limited, as to render possible only the merest smattering of knowledge of the subject. Though insanity is by general consent one of the most pitiable and dreadful of maladies, and though its study is perhaps the most difficult subject to which the mind of man can be addressed, yet it occupies the most insignificant position in the medical curriculum. This fact is not adduced in any way as a reproach to those who regulate and apportion the curriculum. Other factors must be considered by them beyond the terrible character of a disease and the difficulties that its study present. They must take into account also the frequency or rarity of a disease. It would be manifestly disproportionate to allot as much of the student's time to the study of Addison's disease or of insular sclerosis as to that of the treatment of wounds or the complications of labour. Insanity is a terrible malady, it is true, but it is comparatively rare. A medical man in a very large practice may not see one case among his private patients in a year. For this reason the deficiency of his hospital training is not, in this department, compensated by his opportunities in after life. To the great majority of medical practitioners insanity remains as foreign to their experience and knowledge as beri-beri or guinea worm; so that when they are called upon to deal with a case they are sorely put to it to know how to act; they are obliged to call in outside assistance, and they have not even that modicum of knowledge which should enable them to appraise the quality of the assistance on which they rely. Another unfortunate consequence of the general lack of knowledge, not only among medical practitioners, but also among the public at large, is the under-estimation, or even the non-recognition, of the signs of insanity in its early stages and in its slighter manifestations.

When a case of insanity is recognised as such, the difficulty of deciding how it ought to be dealt with is often very great. In the great majority of cases it soon becomes manifest that the patient cannot remain at home. He needs control, safeguarding, or skilled treatment. Whither is the patient to be removed?

The answer to this question must depend on various considerations, the chief of which are the means of the patient and the view taken by the consultant, for in almost every case, not being a pauper case, the services of a consultant are invoked. It happens so very often that the consultant is a neurologist, and has had no special experience of insanity, the alternatives placed before the patient and his friends are usually three—a sea voyage, a nursing home, or residence in the house of a doctor. To these is sometimes added the expedient of travelling on land with an attendant, medical or non-medical. All these methods accomplish very effectually the aim of getting the patient removed from his home. That is in many cases a great relief to the friends; but it is not of itself a curative measure. If he were left to himself he would in many cases attain the same end in order to satisfy his wandering or other propensities. On shipboard the circumstances are not such as we should *prima facie* expect to be very beneficial to a case of incipient insanity, especially to a case of mental depression, and it is for such cases that this course is usually advised. Life on board ship is, by its monotony and want of interest, in itself depressing to many travellers, and it is rarely that a ship's doctor has had any more experience of insanity than other general practitioners. We see frequent evidences of the non-success of this mode of treatment in the newspaper paragraphs which chronicle the accidents of persons when taking sea voyages for their health.

Nursing homes do not offer the same daily and hourly temptations to suicide that are furnished by a sea voyage; but nursing homes are not equipped in the very exceptional manner that has to be followed in institutions for the insane, their staff is not accustomed to deal with the insane, and the result of sending cases of mental disease to nursing homes is not such as to encourage the practice.

All that has been said of nursing homes applies to a majority of those doctors who receive mental cases in single care. In many instances the home is luxurious, the host kind and attentive, his wife perhaps a trained nurse; he has pleasant grounds and a carriage for the use of his patient; but he has had little or no experience of mental disorder, and would himself admit that he is not competent, nor his house adapted, for the reception and treatment of a case of much severity. He is not skilled in the observation and detection of ominous symptoms, and his house is more a place of rest and recreation for an overwrought person than a place of treatment for a case of mental disease. There are many cases of slight mental disorder that are suited for private care in the house of a medical man, but very many cases that are most unsuitable for such care are sent into the private care of medical and non-medical men and women as single patients under certificates, or, much more often, without certificates, although they might easily be, and ought to be, certificated. The very large number of cases so cared for is a great scandal. If the law is unjust and detrimental to the welfare of the persons whom it purports to protect, it should be altered; but as long as it exists, it is surely improper that it should be frequently evaded and nullified.

